# Long COVID-related blood-brain barrier breakdown and microstructure in older adults are modified by sex and Alzheimer’s disease genetic risk

**DOI:** 10.1162/IMAG.a.23

**Published:** 2025-05-28

**Authors:** Emilie T. Reas, Austin Alderson-Myers, Seraphina K. Solders, Qian Shen, Charlotte S. Rivera, Xin Wang, Jordan Stiver, Sarah J. Banks, Jennifer S. Graves

**Affiliations:** Department of Neurosciences, University of California, San Diego, La Jolla, CA, United States

**Keywords:** Alzheimer’s disease, COVID-19, sex differences, blood-brain barrier, diffusion MRI

## Abstract

Long COVID is characterized by lingering symptoms following SARS-CoV-2 infection, which may include neurological and cognitive complaints. Hypothesized mechanisms, including blood-brain barrier (BBB) dysfunction and neuroinflammation, are shared with Alzheimer’s disease (AD) and related dementias. To address concern that long COVID may accelerate cognitive decline and neurodegeneration, this study examined neuroimaging-based markers of BBB breakdown and brain microstructure among older adults with long COVID, and modification by AD risk factors. Individuals with persistent cognitive complaints following SARS-CoV-2 infection (neurological long COVID, NLCV) and cognitively normal controls (50–90 years, 61% women) underwent neuropsychological evaluation, genotyping, dynamic contrast-enhanced MRI to measure BBB permeability, and multi-compartment diffusion MRI to measure brain microstructure. Cognitive and brain measures were compared between NLCV and controls using analysis of covariance, and associations among measures were assessed using linear regression. Interaction models probed modification by sex and AD genetic risk, quantified with a polygenic hazard score. Compared to controls, NLCV exhibited cognitive impairment, BBB breakdown, and subcortical microstructural abnormalities. NLCV-related BBB leakage was widespread across the brain and more pronounced among men, whereas white matter and subcortical microstructural differences were stronger among women. AD polygenic hazard score modified associations of BBB permeability with memory and microstructure, such that higher caudate BBB permeability correlated with worse immediate recall, and higher white matter permeability correlated with higher free water only for those with elevated genetic risk. BBB dysfunction and microstructural compromise may contribute to cognitive symptoms of long COVID in older adults. Sex-specific patterns, and more deleterious associations between brain and memory abnormalities among individuals with elevated AD genetic risk, highlight the need for precision medicine diagnostic and therapeutic approaches for long COVID.

## 1 Introduction

COVID-19 has had a profound public health impact, with an estimated 6.9% of Americans experiencing long COVID, characterized by emergent health complications that persist over a prolonged post-infection period (Fang et al., 2024;Munblit et al., 2022). Among its heterogeneous manifestations, neurological symptoms present in approximately one third of COVID-19 survivors during the year following infection (Giussani et al., 2024). Long COVID is most prevalent in midlife (Fang et al., 2024), a critical period during which modifiable factors may shape cognitive aging trajectories (Whitmer et al., 2005). The potential for post-COVID brain dysfunction to intersect with age-related neuropathology to accelerate cognitive decline, demands urgent investigation to clarify the underpinnings of long COVID-related neurophysiological changes in our aging population.

SARS-CoV-2 may dysregulate brain function via various mechanisms, including direct neuroinvasion and infection, microvascular injury, ischemia, or aberrant immune and neuroinflammatory response (Pandharipande et al., 2023). Postmortem viral RNA persistence (Stein et al., 2022), and*in vitro*evidence for SARS-CoV-2 replication and translocation across brain capillary endothelial-like cells (Krasemann et al., 2022) support the neuroinvasion hypothesis. However, other studies of COVID-19 patients observed leakage of blood-derived proteins into brain tissue in the absence of viral RNA (Song et al., 2023), and elevated cerebrospinal fluid (CSF)/plasma albumin with no detectable CSF SARS-CoV-2 RNA (Bellon et al., 2020), pointing to indirect mechanisms involving blood-brain barrier (BBB) disruption. Despite postmortem evidence for cerebral microvascular and endothelial cell damage (Lee et al., 2021;Sashindranath & Nandurkar, 2021),*in vivo*human studies linking BBB damage to persistent neurological symptoms of COVID-19 remain limited. Elevated plasma matrix metalloproteinase 9, which can disrupt BBB function, has been observed in patients with severe acute COVID-19 (Bonetto et al., 2022). One small study applying dynamic contrast-enhanced (DCE) MRI, which permits topographical measurement of BBB permeability, identified BBB leakage and cortical atrophy in 11 women with long-COVID-related brain fog (Greene et al., 2024).

BBB dysfunction may also promote Alzheimer’s disease (AD) (Andjelkovic et al., 2023), with potential mechanisms including impaired perivascular clearance of amyloid-β and tau (Harrison et al., 2020), dysregulated cerebral blood flow or neurovascular coupling, or promoting a neuroinflammatory environment. Several overlapping pathophysiological changes have been observed following SARS-CoV-2 infection (Pszczołowska et al., 2024), including tau hyperphosphorylation, elevated CSF amyloid-β pathology, and white matter microglial reactivity that elicits neuroinflammation (Fernández-Castañeda et al., 2022;Reiken et al., 2022;Ziff et al., 2022). Further supporting synergy between COVID-19 and AD-related pathophysiology, epidemiological studies have reported increased risk of AD and other cognitive disorders 1 year following COVID-19 (Xu et al., 2022).

Limited evidence suggests that AD risk factors such as sex or genetics modify COVID-19 outcomes. Both AD and long-COVID are more prevalent among women (Fang et al., 2024;Ferretti et al., 2018;Sylvester et al., 2022), whereas men experience more severe acute COVID-19 and higher mortality (Gomez et al., 2021).*APOE4*, the strongest genetic risk factor for sporadic AD, is associated with BBB breakdown (Montagne et al., 2020) as well as COVID-19 severity and mortality (Kuo et al., 2020;Safdari Lord et al., 2022). However, polygenic variants beyond*APOE4*confer AD risk, highlighted by the improved prediction of AD pathology and clinical outcomes by polygenic risk estimates such as the AD polygenic hazard score (PHS) (Reas et al., 2023;Tan et al., 2018). Evaluating long COVID outcomes relative to sex and AD polygenic profiles may optimize risk assessment approaches for neurological sequelae of COVID-19.

Structural MRI studies of COVID-19 survivors have identified brain atrophy in AD-vulnerable regions (Douaud et al., 2022), that correlates with CSF markers of BBB disruption (Sanabria-Diaz et al., 2022). Diffusion MRI studies have reported markers of white matter damage in individuals with persistent COVID-19-related symptoms that are associated with cognitive deficits (Bispo et al., 2022;Boito et al., 2023;Caroli et al., 2023;Paolini et al., 2023), although conflicting findings have been reported (Lu et al., 2020). Multi-compartment diffusion MRI models, such as restriction spectrum imaging (RSI), which dissociates diffusion rate and orientation characteristic of distinct cellular properties (White et al., 2013), may help to identify more nuanced cytoarchitectural abnormalities and resolve prior inconclusive findings.

Thus, more rigorous evaluation of candidate neurobiological pathways by which long COVID precipitates cognitive decline, including BBB dysfunction and subtle microstructural brain abnormalities, will be pivotal for identifying therapeutic targets to restore brain function among those most vulnerable to cognitive impairment. This study applied DCE MRI and RSI to evaluate BBB breakdown and brain microstructure in older adults with persistent COVID-19-related cognitive dysfunction, henceforth referred to as neurological long-COVID (NLCV). To interrogate interactions with AD risk, we also assessed modifying effects of sex and the AD PHS, on NLCV-related BBB permeability and brain microstructure.

## 2 Methods

### 2.1 Participants

Participants were recruited from the broader San Diego community (*N*= 35) and the UC San Diego Shiley-Marcos Alzheimer’s Disease Research Center (ADRC;*N*= 44). Participants were considered to have NLCV if 1) they met criteria for long COVID, defined by the World Health Organization as the continuation or development of new symptoms for at least 3 months following a SARS-CoV-2 infection (Soriano et al., 2022), 2) symptoms included persistent cognitive or other neurological complaints (The Lancet, 2021), and 3) they reported no history of pre-existing cognitive impairment. Cognitively normal (CN) participants from the ADRC were diagnosed by two senior neurologists and a neuropsychologist, according to ADRC protocol following clinical, neurological, and neuropsychological evaluation (Galasko et al., 1994). Community participants completed neuropsychological evaluation and were considered CN if they reported no subjective cognitive complaints and demonstrated no objective impairment on the mini-mental state exam (MMSE) (community CN MMSE scores were ≥29). Exclusion criteria included cognitive impairment unrelated to COVID-19, history of stroke, other neurological disease, substance use disorder, MRI safety contraindication, kidney disease, glomerular filtration rate <60 mL/min/1.73 m^3^, or allergy to gadolinium-based contrast agents. One CN and two NLCV participants were excluded from analysis for a prior concussion, an abnormal MRI finding, and a technical issue during MRI acquisition (Supplementary Fig. 1). The final sample included 49 CN (51% women; age: mean ± SD 75.1 ± 7.6, range 51–90 years) and 27 NLCV (78% women; age: 61.6 ± 9.6, 50–81 years) participants.

### 2.2 Standard protocol approvals and participant consents

Study procedures were approved by the University of California, San Diego Human Research Protections Program Board, and participants provided informed written consent prior to participation.

### 2.3 COVID-19 history

A standardized questionnaire assessed history of SARS-CoV-2 infection, vaccination, and post-viral symptoms, including date of SARS-CoV-2 infection, and for multiple infections, the infection date after which post-viral symptoms began. Duration of post-viral condition was computed as time between infection and MRI. Hospitalization for COVID-19 was reported (no/yes) and acute COVID-19 severity was rated on a 0–3 scale (0 = asymptomatic, 1 = mild, 2 = moderate, 3 = severe). For analysis, participants were categorized as mild/moderate versus severe (no participants reported asymptomatic infections). To evaluate post-viral symptoms, participants rated the severity of change since COVID-19 illness in 23 symptoms (0 = none to 10 = severe).

### 2.4 Cognitive assessment

A comprehensive neuropsychological test battery (Salmon & Butters, 1992) was administered by a trained examiner in a quiet room. Specific neuropsychological variables of interest included the MMSE, a cognitive screening tool that tests global cognition. The Trail-Making Test, Part B measures time to complete a letter-number sequencing task and assesses processing speed and executive function. Animal naming evaluates semantic verbal fluency, and requires participants to name as many unique animals as possible within 1 min. The California Verbal Learning Test–Second Edition (CVLT-II) evaluates verbal recall from a list of categorized words; this study analyzed measures of learning (trials 1-5 correct) as well as immediate and delayed free recall. Participants were classified as cognitively unimpaired or having mild cognitive impairment (MCI) based on the Jak/Bondi actuarial neuropsychological criteria (Jak et al., 2009). MCI classification was omitted for one NLCV because the Trails B was not administered, due to pandemic testing limitations.

### 2.5 AD PHS computation

For community participants, genetic sequencing was conducted on saliva samples by Diagnomics, Inc. using the Illumina V2.2 array. For ADRC participants, genetic data were provided by the National Alzheimer’s Coordinating Center (NACC). The AD PHS was developed to estimate polygenic risk for AD and is associated with age of AD onset (Desikan et al., 2017). AD PHS computation was performed by Diagnomics, Inc by combining 198,424 single nucleotide polymorphisms with two*APOE*variants (*ε2/ε4*). Participants were classified as having a high or low PHS if their score was above (positive) or below (negative) the population 50^th^percentile, respectively.

### 2.6 Imaging data acquisition

Imaging data were acquired on two 3.0 Tesla Discovery 750 scanners (GE Healthcare, Milwaukee, WI, USA) at the University of California, San Diego Center for Translational Imaging and Precision Medicine. Following a three-plane localizer, a sagittal 3D fast spoiled gradient echo (FSPGR) T_1_-weighted structural scan optimized for maximum tissue contrast (TR = 6.7 ms, inversion time = 450 ms, flip angle = 8°, FOV = 240 × 240 mm, matrix = 256 × 256, slice thickness = 1.2 mm, resampled to 1 mm^3^resolution), and an axial 2D single-shot pulsed-field gradient spin-echo echo-planar diffusion-weighted sequence (45 gradient directions, b-values = 0, 500, 1500, 4000 s/mm^2^, one b = 0 volume and 15 gradient directions for each non-zero b-value; TR = 8 s, FOV = 240 × 240 mm, matrix = 96 × 96, slice thickness = 2.5 mm, resampled to a 2 mm^3^resolution) were acquired. For BBB permeability measurement, four FSPGR sequences (20 s each) with flip angles of 2, 5, 10, 15 (all other parameters equal to the subsequent DCE FSPGR) were conducted for T_1_-mapping, followed by an axial DCE FSPGR sequence (flip angle = 30°, FOV = 256 × 256 mm, matrix = 256 × 256, slice thickness = 5 mm, slices = 28). Due to software upgrades, DCE image volumes ranged from 53–58, TR ranged from 7.7–8.2 ms, and temporal resolution ranged from 18.3–20.3 s. After a 3-min baseline, Gadavist (gadobutrol; 0.1 mL/kg) was injected at a flow rate of 2 mL/s, followed by a 20 mL saline flush.

### 2.7 Data processing

After visual inspection of raw and processed MR images, data were processed using the Multi-Modal Processing Stream (Hagler Jr et al., 2019), an automated image processing pipeline that integrates FreeSurfer and FMRIB Software Library (FSL) with in-house software. Cortical gray matter, white matter, and CSF boundaries were reconstructed from T_1_-weighted images using FreeSurfer (version 6.0), and subcortical regions were automatically segmented according to a subcortical atlas (Fischl et al., 2002). Cortical editing to remove non-brain voxels or add white matter control points was conducted as necessary. Diffusion MRI data underwent eddy current correction, correction for head motion with rigid-body registration, and correction for B_0_field inhomogeneity spatial and intensity distortions, as previously detailed (Hagler Jr et al., 2019). The b = 0 images were registered to T_1_images using mutual information, and diffusion images were aligned with a fixed rotation and translation relative to the T_1_image. White matter tracts were labeled using AtlasTrack, a fiber atlas based on prior probability and orientation information (Hagler et al., 2009), and voxels containing primarily gray matter or CSF were excluded from white matter (Fischl et al., 2002). DCE FSPGR images were registered to T_1_images using mutual information coregistration in SPM12 (http://fil.ion.ucl.ac.uk/spm/).

### 2.8 Computation of imaging metrics

K_trans_, the transfer coefficient reflecting neurovascular permeability, was computed from DCE MRI images in ROCKETSHIP (Barnes et al., 2015) using the Patlak model, which is optimal for conditions of low-permeability (Heye et al., 2016). Hematocrit levels were measured from blood samples collected within 6 weeks before MRI. A vascular input function was derived from the superior sagittal sinus, recommended to reduce partial volume effects and inflow artifacts (Ewing et al., 2003), using the 3D Fill tool in MRIcron (https://www.nitrc.org/projects/mricron). Using the participant-specific volume with peak vessel signal intensity, the origin was set in the vessel center along the posterior parietal midline, the radius was set to 50 mm, and other settings were adjusted to ensure vessel coverage while avoiding pixels at the edge. To exclude physiologically implausible values, K_trans_was thresholded using a minimum value of 10^-7^.

Diffusion data were fit with the RSI model (Hagler Jr et al., 2019;White et al., 2013). RSI applies linear estimation allowing combinations of restricted, hindered, and free water diffusion, with separate fiber orientation density (FOD) functions (4^th^order spherical harmonic), permitting multiple volume fractions and diffusion orientations within a single voxel. Each measure was normalized by computing the Euclidean norm of the corresponding model coefficients divided by the norm of all coefficients. Computed RSI metrics included: restricted isotropic diffusion, the fraction of intracellular diffusion present in cell bodies; neurite density, anisotropic restricted diffusion consistent with neurites; hindered diffusion, non-restricted isotropic diffusion that is hindered by cellular barriers and is consistent with diffusion within large cell bodies or the extracellular space; and free water, a measure of CSF. Restricted isotropic and hindered diffusion were computed from the 0^th^order spherical harmonic coefficients of the restricted and hindered fractions, respectively; neurite density was derived from the 2^nd^and 4^th^order spherical harmonic coefficients of the restricted fraction. Due to an MRI scanner software upgrade that affected the diffusion MRI sequence, RSI metrics were adjusted for scanner software version by computing the regression residual.

For*a priori*analyses, imaging metrics were computed across global gray and white matter, and within six subcortical regions (hippocampus, thalamus, caudate, putamen, amygdala, nucleus accumbens). Measures within regions of interest were averaged between hemispheres, and global metrics were computed as the mean signal across the FreeSurfer-derived cortical gray matter mask and AtlasTrack-derived fiber tracts, respectively. For post-hoc regional analysis of white matter, measures were computed within 15 fiber tracts.

### 2.9 Statistical analysis

Analyses were conducted in SPSS version 29.0 (IBM Corp, Armonk, NY, USA), FreeSurfer (version 6.0), and FSL (version 5.0.2.2). Significance was set to*p*< 0.05. Analyses of subcortical regions used a Bonferroni-corrected threshold of*p*< 0.008 to account for multiple comparisons. For any significant effects across global white matter, post-hoc analyses of individual white matter tracts were conducted using an adjusted threshold of*p*< 0.003 to account for comparison across multiple tracts. Significance for interaction models was set at an unadjusted threshold of*p*< 0.05. Voxel-wise analyses were corrected using the family-wise error method.

Differences in participant characteristics between NLCV and CN, or according to COVID-19 severity, were examined using two-tailed independent-samples*t*-tests for continuous variables or chi-squared tests for categorical variables. Differences in cognitive function between NLCV and CN were tested using analysis of covariance (ANCOVA), adjusted for age, sex, and education. To examine group differences in BBB permeability or brain microstructure, ANCOVA were conducted with K_trans_or RSI metric as the dependent variable and covariates of age and sex. Differences in cognitive function, BBB permeability, and microstructure were compared between NLCV with mild to moderate and severe COVID-19. To evaluate effect modification of NLCV-related cognitive impairment, BBB breakdown, or microstructural abnormalities by sex or PHS, ANCOVAs were repeated, as described above, with a term for the multiplicative interaction between group and sex or PHS. Significant interactions were followed by sex- or PHS-stratified analyses.

To evaluate whether NLCV-related cognitive function was associated with BBB permeability or microstructure, linear regressions were conducted with cognitive test score as the outcome, K_trans_or RSI metric as the regressor, a term for the interaction between group and imaging metric (mean centered), and covariates of age, sex, and years of education. Significant interactions were followed by regressions stratified by group. Regressions were similarly conducted among NLCV to assess interactions with sex or PHS, followed by sex- or PHS-stratified models.

To assess associations between BBB breakdown and proximal microstructure, linear regressions were performed with K_trans_as the regressor, RSI metrics as the outcome, and covariates of age and sex. Regressions were conducted, as described above, with interactions between K_trans_and group, or within NLCV with interactions between K_trans_and sex or PHS.

For post-hoc whole-brain voxel-wise analysis of group differences, a study-specific anatomical template was created using Advanced Normalization Tools (ANTs;http://stnava.github.io/ANTs/) to maximize the accuracy of registration to a common space. An initial template registered representative anatomical scans from five CN to MNI152 1 mm space using a linear registration with 12 degrees of freedom (FSL’s FLIRT) (Jenkinson & Smith, 2001), then averaged to create a mean anatomical image used as the initial template in ANTs multivariate template construction, derived from fifty-two anatomical scans (from a representative sample of older adults across the cognitive continuum). All anatomical scans were registered to the group template, and this registration was applied to K_trans_or RSI maps. Voxel-wise analyses of K_trans_or RSI maps were performed using randomize in FSL with 5000 permutations and the threshold-free cluster enhancement (TFCE) test statistic.

## 3 Results

### 3.1 Participant characteristics

Participant characteristics are shown inTable 1. NLCV were younger and had a greater proportion of women and Hispanic or non-White participants (*p*< 0.05) than CN. Education level and the proportion of high-PHS individuals did not differ between groups (*p*> 0.40).

**Table 1.** Participant characteristics (mean ± SD^a^or*N*(%)) for neurological long COVID (NLCV) and cognitively normal (CN) participants.

	*N*	CN *N* = 49	NLCV * N* = 27	Group difference
Age (years) [range]	76	75.1 ± 7.6 [51-90]	61.6 ± 9.6 [50-81]	***t*** **(74) = 6.78,** ***p*** **<** **0.001**
Sex (women)	76	*N* = 25 (51%)	*N* = 21 (78%)	***x*** **^2^ (1) = 5.22, ** ***p*** **=** **0.02**
Education (years)	76	16.9 ± 2.1	16.4 ± 2.0	*t* (74) = 0.73, *p* = 0.44
Non-Hispanic White	76	*N* = 47 (96%)	*N* = 20 (74%)	***x*** **^2^ (1) = 7.96, ** ***p*** **=** **0.005**
High PHS (>50 ^th^ percentile)	51	*N* = 14 (52%)	*N* = 10 (42%)	*x* ^2^ (1) = 0.53, *p* = 0.47
Time between MRI and cognitive testing (months)	76	0.4 ± 7.2	0.4 ± 0.6	*t* (74) = 0.02, *p* = 0.99
MMSE	76	29.1 ± 1.2	28.4 ± 1.8	*F* (1,71) = 2.51, *p* = 0.12
Trails B	75	76.7 ± 31.4	91.4 ± 33.3	*F* (1,70) = 2.48, *p* = 0.12
Semantic fluency	76	23.1 ± 4.6	19.0 ± 5.9	***F*** **(1,71) = 7.03,** ***p*** **=** **0.01**
CVLT-II learning	76	49.1 ± 11.3	41.5 ± 11.8	***F*** **(1,71) = 5.05,** ***p*** **=** **0.03**
CVLT-II immediate recall	76	10.9 ± 3.7	8.5 ± 3.3	***F*** **(1,71) = 5.25,** ***p*** **=** **0.02**
CVLT-II delayed recall	76	11.3 ± 3.8	8 *.9* ± 3.8	***F*** **(1,71) = 5.18,** ***p*** **=** **0.03**

MMSE, mini-mental state exam; CVLT-II, California verbal learning test – second edition. Cognitive test scores are adjusted for age, sex, and education. Bold indicates significant (*p*< 0.05).

Among NLCV, mean interval between SARS-CoV-2 infection and MRI was 1.74 ± 1.07 (range 0.32–4.15) years. Four, 9, and 14 participants reported mild, moderate, and severe acute COVID-19 illnesses, respectively, and three were hospitalized for acute infections. Illness severity was unrelated to age, sex, education, or PHS. Loss of smell and taste were reported in 59% and 67% of NLCV, respectively, with 26% persistence beyond 3 months. Among NLCV for whom COVID-19 vaccination history was available (*N*= 25), 56% were not vaccinated at symptom onset. Frequently reported post-viral symptoms are presented inSupplementary Figure 2, with the most prevalent including memory difficulties (93%) and trouble concentrating (89%).

### 3.2 NLCV demonstrate cognitive dysfunction

**Fig. 1.**
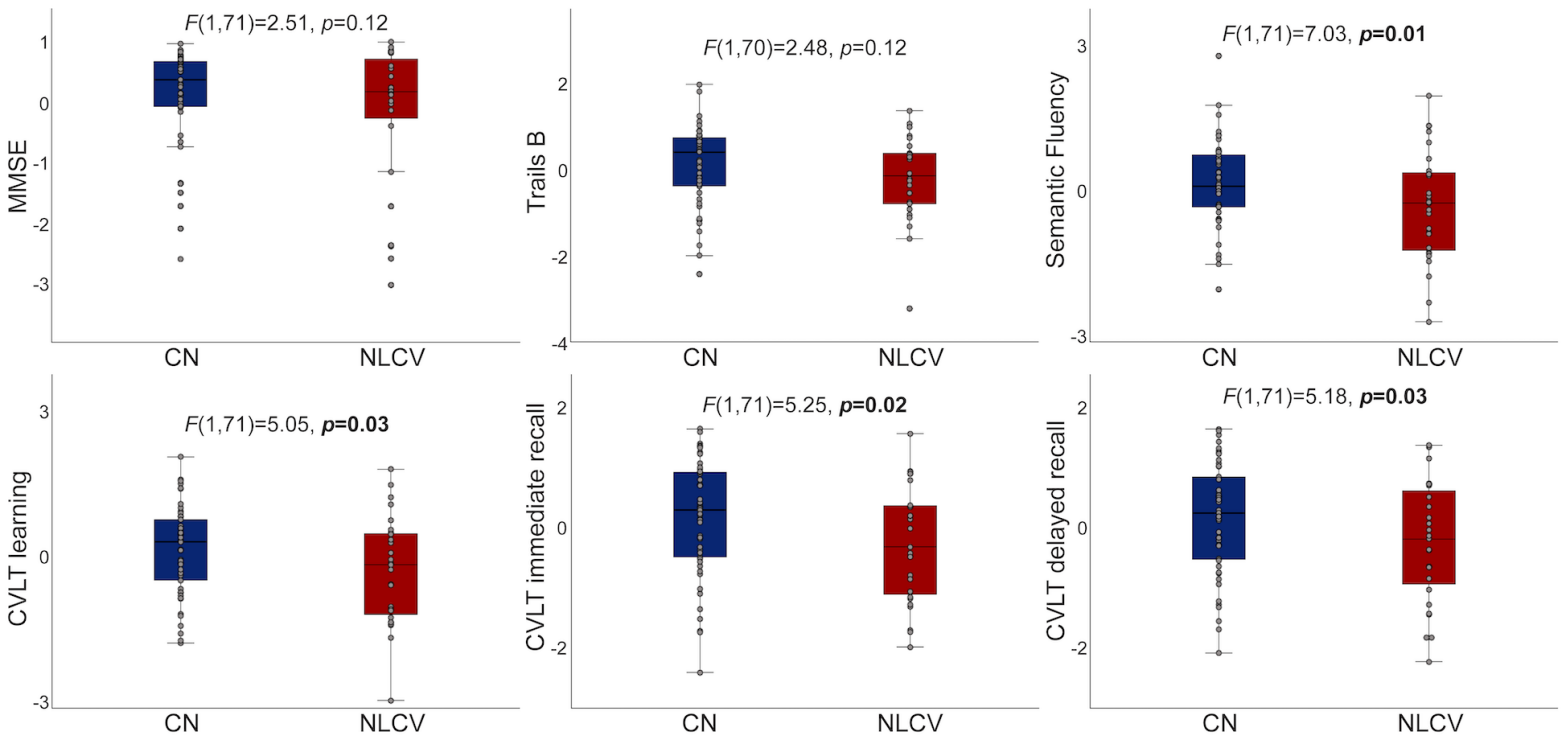
Differences in cognitive function between cognitively normal and neurological long Covid. Cognitive test scores are shown for cognitively normal (CN) and neurological long COVID (NLCV). The y-axis for Trails B is inverted, such that higher values represent shorter time to completion. Values represent residuals, adjusted for age, sex, and education.

A higher proportion of NLCV (39%) met criteria for MCI than CN (16%) (*x*^2^(1) = 4.56,*p*= 0.03). NLCV did not differ from CN in global cognition or executive function, but performed worse on semantic fluency, as well as verbal learning, immediate recall, and delayed recall (*p*< 0.05, adjusted for age, sex, and education;Table 1,Fig. 1). Compared to those with mild to moderate illness, NLCV with severe COVID-19 performed worse on semantic fluency (*F*(1,22) = 4.74,*p*= 0.04) and verbal immediate recall (*F*(1,22) = 4.69,*p*= 0.04). Longer time since infection correlated with worse executive function (*r*= 0.43,*p*= 0.04).

Although the small sample of NLCV men limited power to examine sex interactions, exploratory analyses revealed a pattern whereby women (*p*< 0.05), but not men (*p*> 0.36), with NLCV performed worse than CN on semantic fluency, learning, and memory (Supplementary Table 1). PHS did not modify differences in cognitive function between CN and NLCV.

### 3.3 BBB breakdown and abnormal brain microstructure among NLCV

**Fig. 2.**
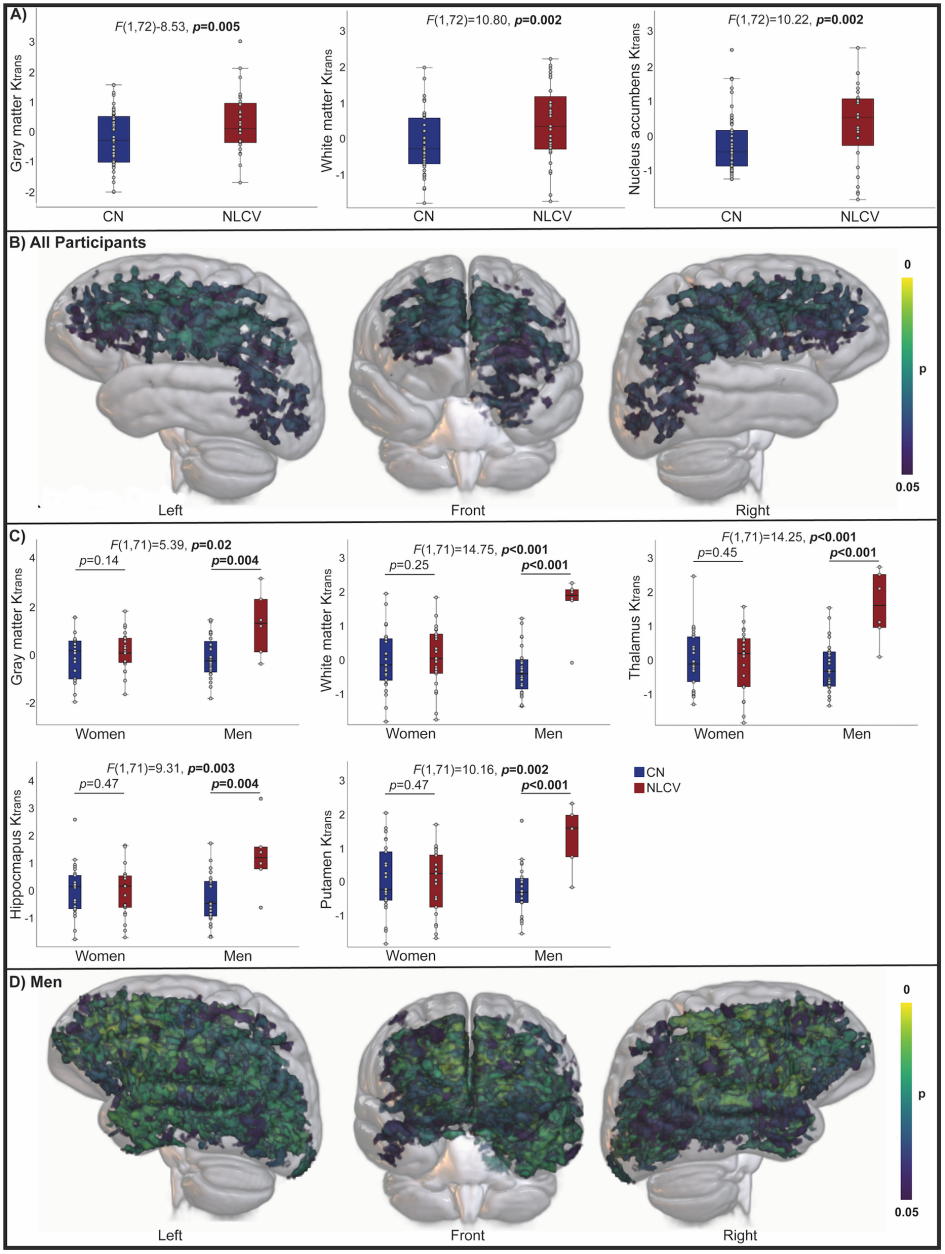
Differences in blood-brain barrier permeability between cognitively normal and neurological long Covid. K_trans_for cognitively normal (CN) and neurological long COVID (NLCV) (adjusted for age and sex) are shown for regions demonstrating significant differences (A). Voxel-wise differences in K_trans_between CN and NLCV for all participants are shown in (B). K_trans_values demonstrating significant sex by group interactions (adjusted for age) are shown in (C), and voxel-wise differences between CN and NLCV for men are shown in (D). Values in A and C represent residuals, adjusted for respective covariates. Voxel-wise maps (B, D) are family-wise error corrected.

K_trans_was higher for NLCV than CN across gray (*F*(1,72) = 8.53,*p*= 0.005) and white (*F*(1,72) = 10.80,*p*= 0.002) matter, and in the nucleus accumbens (*F*(1,72) = 10.22,*p*= 0.002), (adjusted for age and sex;Fig. 2A). Voxel-wise analyses confirmed widespread increases in K_trans_, with most pronounced effects in the bilateral frontal and parietal lobes (Fig. 2B), and tract-specific analysis revealed the strongest differences in frontal association and commissural fibers (Supplementary Table 2). Sex interacted with group for K_trans_in gray matter, white matter, thalamus, putamen, and hippocampus, reflecting higher K_trans_for NLCV than CN among men only (Fig. 2C). Sex-stratified whole-brain analyses identified a more widespread but similar topographic distribution of the NLCV-related increase in K_trans_among men as for the full sample (Fig. 2D).

**Fig. 3.**
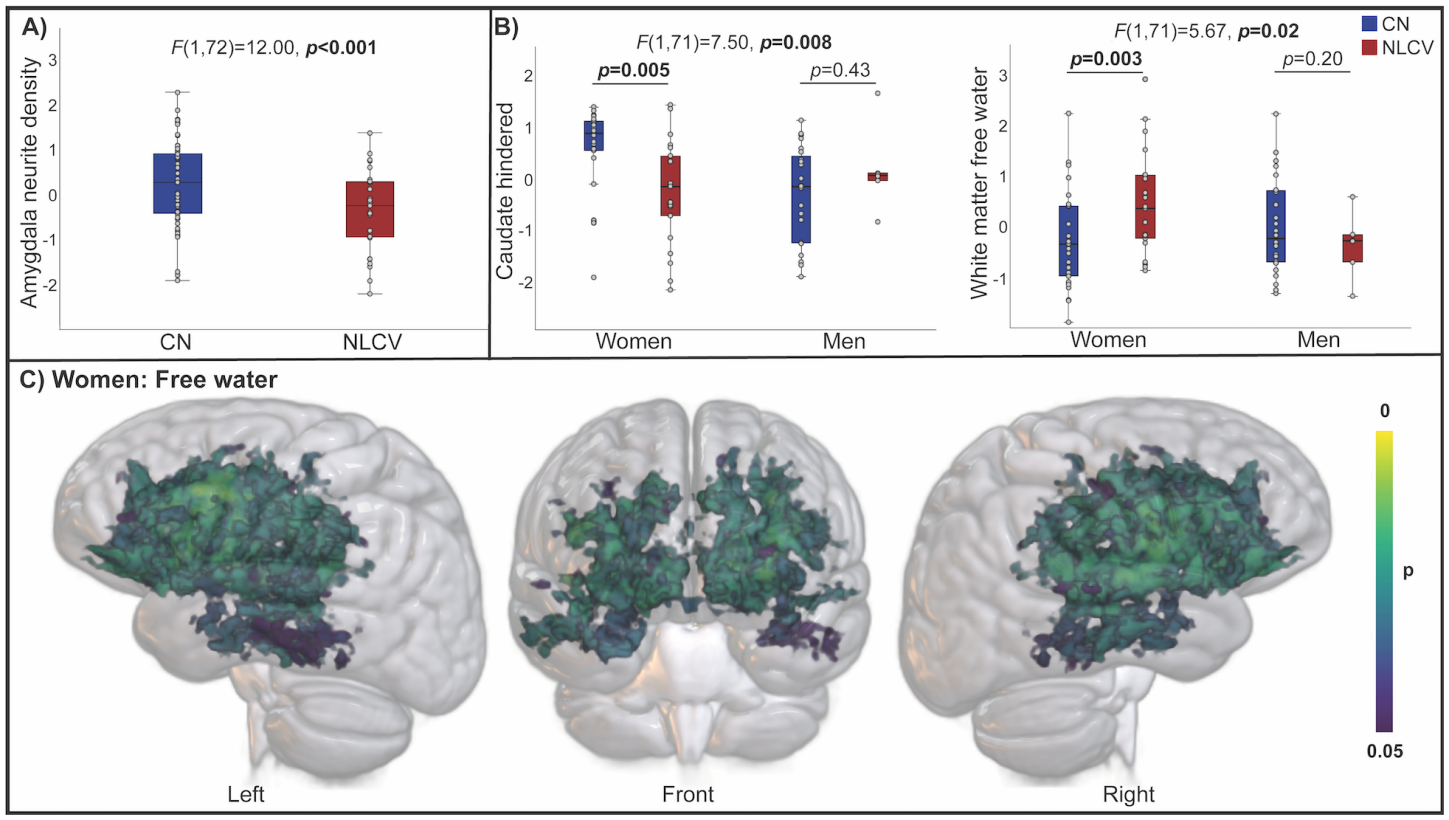
Differences in brain microstructure between cognitively normal and neurological long Covid. RSI metrics demonstrating significant differences between cognitively normal (CN) and neurological long COVID (NLCV) (adjusted for age, sex, and software) are shown in (A). RSI metrics demonstrating significant sex by group interactions (adjusted for age and software) are shown in (B). Voxel-wise differences in free water (adjusted for age and software, family-wise error corrected) between CN and NLCV are shown for women in (C). Values in A and B represent residuals, adjusted for respective covariates.

Amygdala neurite density was lower in NLCV compared to CN (adjusted for age and sex;*F*(1,72) = 12.00,*p*< 0.001;Fig. 3A). Sex-modified NLCV-related differences in white matter free water and caudate hindered diffusion, driven by higher free water and lower hindered diffusion among NLCV women only (Fig. 3B). Sex-stratified voxel-wise analyses revealed increased free water among women NLCV, particularly among frontal white matter (Fig. 3C), and tract-specific analyses demonstrated the strongest differences in frontal projection and association fibers (Supplementary Table 3).

Although all analyses adjusted for age and sex, to further assess whether group differences in these factors influenced findings, sensitivity analyses were conducted using a subset of 27 CN who were more closely matched to NLCV on age (70.1 ± 5.8 years; range 51–76) and sex (56% women). Group differences were not altered, such that significantly higher gray matter, white matter, and nucleus accumbens K_trans_, and lower amygdala neurite density, for NLCV than CN persisted (all*p*< 0.01;Supplementary Table 4).

Neither K_trans_nor microstructure differed by COVID-19 severity. PHS did not modify differences in K_trans_or microstructure between NLCV and CN.

### 3.4 Associations of cognitive function with brain microstructure and BBB permeability

For both CN and NLCV, higher gray matter free water was associated with worse semantic fluency (CN*ß*= -0.37,*p*= 0.03; NLCV*ß*= -0.66,*p*= 0.02) and higher hippocampal free water was associated with worse verbal immediate (CN*ß*= -0.42,*p*= 0.007; NLCV*ß*= -1.11,*p*< 0.001) and delayed (CN*ß*= -0.46,*p*= 0.003; NLCV*ß*= -0.90,*p*= 0.002) recall (Supplementary Table 5). There was a trend for an interaction between group and hippocampal free water for verbal learning, and a significant interaction between group and microstructure for executive function. These effects were driven by stronger correlations between higher free water and worse learning for NLCV, and of lower gray matter restricted isotropic diffusion and higher white matter hindered diffusion with worse executive function for CN. Associations between K_trans_and cognitive function did not differ between CN and NLCV.

Sex did not significantly modify associations of brain microstructure or K_trans_with any cognitive measure. However, there was a trend for an interaction between sex and hippocampal free water on verbal immediate recall, reflecting stronger associations between higher free water and worse recall for women (Supplementary Table 6).

**Fig. 4.**
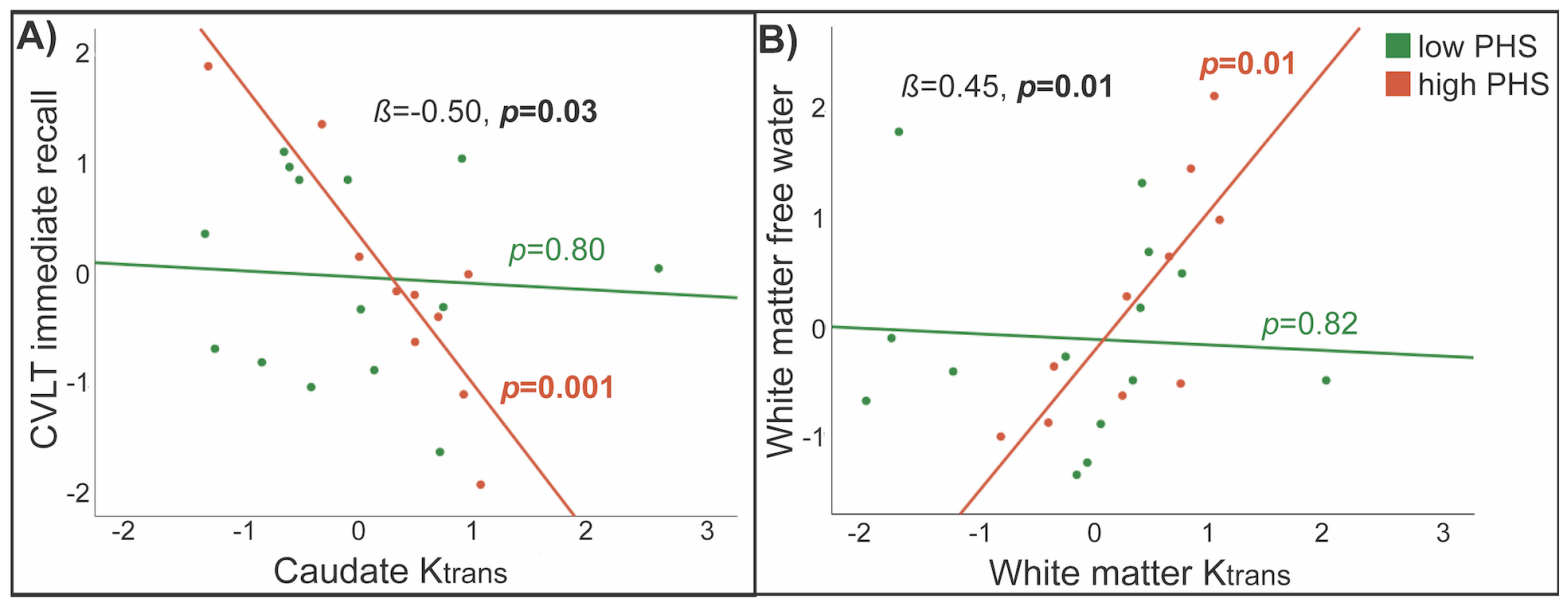
Differences in associations of K_trans_with memory and microstructure by Alzheimer’s disease polygenic hazard score. Among neurological long COVID (NLCV) participants, Alzheimer’s disease polygenic hazard score (PHS) modified associations between caudate K_trans_and immediate recall (A, adjusted for age, sex, and education), and between white matter K_trans_and free water (B, adjusted for age, sex, and scanner software), with correlations present only for NLCV with high PHS. Values represent residuals, adjusted for respective covariates.

There was a significant interaction between PHS and caudate K_trans_for verbal immediate recall (*ß*= -0.50,*p*= 0.03), such that correlations were present only for high-PHS NLCV (*ß*= -1.01,*p*= 0.001;Supplementary Table 7;Fig. 4A). PHS did not modify associations between microstructure and cognition (Supplementary Table 7).

### 3.5 Associations between BBB permeability and brain microstructure

BBB permeability did not correlate with brain microstructure across all NLCV participants. There was a significant interaction between PHS and white matter K_trans_on white matter free water (*ß*= 0.45,*p*= 0.01), reflecting a correlation between higher K_trans_and higher free water among high-PHS only (*ß*= 0.91,*p*= 0.01;Fig. 4B). Sex did not modify associations between K_trans_and any RSI measure.

## 4 Discussion

In this study interrogating cerebrovascular and cytoarchitectural abnormalities in older adults with persistent cognitive complaints following SARS-CoV-2 infection, NLCV presented with objective cognitive impairment, BBB breakdown, and microstructural abnormalities, compared to controls. Men and women exhibited distinct NLCV-related BBB and microstructural differences, and NLCV at elevated genetic risk for AD demonstrated associations of BBB leakage with memory impairment and microstructural injury.

Among individuals with NLCV, BBB permeability was broadly elevated across the cortical gray matter and white matter. This*in vivo*neuroimaging finding supports mounting evidence from biofluid and post-mortem studies implicating BBB dysfunction in the neurological sequelae of COVID-19 (Bellon et al., 2020;Bonetto et al., 2022;Song et al., 2023). The endothelial cell expression of angiotensin-converting enzyme 2, which is the target receptor of SARS-CoV-2 and involved in cardiometabolic conditions including hypertension and diabetes (Batiha et al., 2021), may partially explain this observed vulnerability of the cerebrovasculature to COVID-19. A central role of vascular dysfunction in COVID-19 pathophysiology is further supported by the susceptibility of individuals with pre-existing vascular disorders to more adverse disease outcomes, and increased incidence of post-infection cerebrovascular complications (Sashindranath & Nandurkar, 2021). Our results extend preliminary findings from another DCE MRI investigation reporting frontal-temporal BBB breakdown in a small sample of women with long COVID-related brain fog (Greene et al., 2024) to suggest that BBB disruption in NLCV may be a more widespread, whole-brain phenomenon. Although BBB leakage was diffuse, frontal cortex and association fibers were relatively more affected. Differences in study findings may be attributable to our larger sample powered to detect more subtle leakage, our inclusion of men, or the older age of our participants. Indeed, despite our predominantly female NLCV group, exploratory analyses revealed a sex difference whereby BBB breakdown in NLCV was more pronounced among men. Although we (Reas et al., 2024) and others have not reported reliable associations between K_trans_and age among older adults (Greene et al., 2024;Nation et al., 2019), the possibility that age magnifies effects of long COVID on neurovascular damage warrants investigation.

Across all participants, NLCV presented with reduced neurite density in the amygdala. However, more severe microstructural injury was observed among NLCV women, who exhibited a reduced fraction of hindered diffusion in the caudate and elevated white matter free water. Increased white matter free water was widespread, but more prominent in frontal association and projection fibers, paralleling the frontal distribution of BBB breakdown in men. White matter is particularly vulnerable to small vessel disease and ischemia (Wardlaw et al., 2015), which are associated with neuroinflammation, cellular barrier degeneration due to axon or myelin damage, and altered fluid dynamics, properties that would be captured by the increased free water observed here. Further research will be important to assess potential vascular or neuroinflammatory origins of COVID-19-related white matter injury. Frontal white matter, amygdala, and caudate microstructural damage, which may support executive, attention, emotional salience, and memory networks (Grahn et al., 2008;LeDoux, 2007), may, in part, account for the multi-domain cognitive deficits observed in NLCV. Notably, other studies of COVID-19 patients with neurological or persistent symptoms have also reported widespread (Bispo et al., 2022;Boito et al., 2023;Caroli et al., 2023) or frontally localized (Campabadal et al., 2023) white matter compromise as well as volume loss in regions encompassing the frontal cortex, caudate, and amygdala (Sanabria-Diaz et al., 2022).

These sex differences in patterns of BBB leakage and microstructural damage implicate sex-specific neurological mechanisms of NLCV. It is well appreciated that the manifestations and physiological perturbations from COVID-19 are multifaceted and uniquely individual, with mounting evidence for viral persistence, vascular damage, inflammation, and immune dysregulation, among the variable pathways by which SARS-CoV-2 may disrupt brain health (Pandharipande et al., 2023). Sex differences in vascular risk factors, immune signaling, or hormonal factors, may predispose men and women to distinct complications of COVID-19. For instance, compared to premenopausal women, men are at higher risk for cardiovascular disease and demonstrate differences in endothelial function (Stanhewicz et al., 2018), which may contribute to increased cumulative vascular risk exposure that lowers the threshold for COVID-related cerebrovascular damage. In contrast, the greater propensity for autoimmune or inflammatory conditions among women (Klein & Flanagan, 2016) may predispose them to neuroinflammation and immune dysregulation with long COVID.

Brain microstructure correlated with cognitive function, with a pattern of stronger associations among CN for executive function and among NLCV for memory. NLCV-specific correlations were particularly pronounced for free water in the hippocampus, which is critical for the formation of episodic memories and a target of AD-related neurodegeneration (Gosche et al., 2002) that contributes to the characteristic amnestic deficits that manifest early in typical AD. Our findings point to distinct microstructural fingerprints of cognitive dysfunction associated with aging and long COVID, specifically implicating cytoarchitectural hippocampal vulnerability to long COVID that may account for corresponding memory decline. Further research is warranted to assess the long-term implications of this observation, including whether hippocampal compromise from NLCV may hasten neurodegenerative changes in AD or related disorders.

Within NLCV, only those individuals at heightened genetic risk for AD exhibited associations between caudate BBB breakdown and impaired immediate recall, and between white matter BBB leakage and higher free water. The caudate is structurally and functionally connected with the prefrontal cortex, subserving goal-directed behavior (Grahn et al., 2008), such that neurovascular dysfunction in the caudate may disrupt circuitry crucial for the attentional and executive demands of memory recall. A link between white matter BBB leakage and free water specific for high-PHS NLCV points to AD-dependent mechanisms by which neurovascular dysfunction may promote white matter damage. Our findings raise the disconcerting possibility that BBB damage associated with long COVID is particularly deleterious for individuals with genetic predisposition to AD, potentially contributing to memory decline and widespread white matter injury. Synergy between the pathophysiology of AD and long COVID is supported by evidence for shared genetic vulnerability to COVID-19 and AD, as well as common mechanistic pathways including microglia-mediated neuroinflammation, tau hyperphosphorylation, and amyloid-β accumulation (Golzari-Sorkheh et al., 2023), underscoring the need for further research into the effects of long COVID on the precipitation of AD and related dementias.

Strengths of this study include the application of advanced MRI approaches to quantify neurovascular permeability and brain microstructure, a focus on older adults, and examination of dementia risk factors to probe synergy between long COVID and AD pathophysiology, all of which have been minimally examined in the context of long COVID. Although participants were well-characterized in terms of illness severity, hospitalization, vaccination, and post-acute symptoms, this study was not designed to assess subgroup differences. Considering the diversity of long COVID symptom presentation and corresponding variability in underlying mechanisms, larger studies powered to probe differences by NLCV subtype are needed to guide precision-medicine therapeutic strategies. Our NLCV sample was disproportionately female, which is representative of the population and consistent with the established female disadvantage for long COVID (Fang et al., 2024), yet limited power to interrogate sex differences; thus, the sex differences reported here should be considered hypothesis-generating and validated in larger samples. Although CN and NLCV differed in age and sex, results were unchanged in sensitivity analyses using a subset of more closely matched CN participants, suggesting that these group differences did not materially affect our results. While our findings implicate an adverse modifying effect of AD genetic risk on NLCV-related brain outcomes, longitudinal investigations incorporating AD biomarkers will be important to establish the long-term effects of COVID-19 on AD pathogenesis among high-risk populations.

In summary, we report BBB breakdown and microstructural brain abnormalities among older adults with persistent cognitive complaints subsequent to SARS-CoV-2 infection. Sex-specific patterns highlight the heterogeneity of mechanisms underlying neurological long COVID and underscore the need for precision-medicine diagnostic and treatment approaches. Magnified associations of BBB leakage with worse cognitive and microstructural outcomes among NLCV with high AD genetic risk bolsters concern for a “perfect storm” of synergistic long COVID and AD pathophysiology, warranting urgent investigation into the long-term impact of COVID-19 on the pathogenesis of AD and related dementias.

## Supplementary Material

Supplementary Material

## Data Availability

Data that support the findings of this study are available within this article and itsSupplementary material. Raw data supporting the findings of this study and code used for analysis are available from the corresponding author upon reasonable request.
