# Supplementary material for: Long COVID-related blood-brain barrier breakdown and microstructure in older adults are modified by sex and Alzheimer’s disease genetic risk
Source: Imaging Neurosci (Camb). 2025 May 28;3:IMAG.a.23. doi: 10.1162/IMAG.a.23 (PMC12319845; doi:10.1162/IMAG.a.23)

Supplementary Data:

Long COVID-related blood-brain barrier breakdown and microstructure in older adults are modified by sex and Alzheimer's disease genetic risk

Supplementary Table 1. Differences in cognitive test scores between neurological long COVID (NLCV) and cognitively normal (CN) participants (adjusted for age and education), are presented for women and men, along with sex by group interactions.

	**WOMEN**		**MEN**		
**Tract**	**CN**	**NLCV**	**CN**	**NLCV**	**Sex interaction**
	***N*=25**	***N*=21**	***N*=24**	***N*=6**	
MMSE	29.2±1.1	28.6±1.9	28.9±1.3	28.0±1.8	$F(1,70)=0.08$, $p=0.78$
Trails B	74.3±29.1	91.6±36.3	79.5±34.1	89.1±22.7	$F(1,69)=0.21$, $p=0.65$
Semantic fluency	**24.3±4.3**	**19.2±6.2***	21.5±4.7	19.6±5.1	$F(1,70)=1.47$, $p=0.24$
CVLT-II learning	**53.3±8.5**	**43.6±12.3***	43.1±12.1	40.1±10.3	$F(1,70)=1.26$, $p=0.27$
CVLT -II immediate recall	**12.2±2.6**	**9.1±3.5****	9.3±4.3	8.0±2.5	$F(1,70)=0.90$, $p=0.35$
CVLT-II delayed recall	**12.5±2.9**	**10.0±3.5***	9.5±4.2	7.2±4.1	$F(1,70)=0.01$, $p=0.91$

MMSE, mini-mental state exam; CVLT-II, California verbal learning test - second edition

* $p<0.05$, ** $p<0.01$ between NLCV and CN within sex

Supplementary Table 2. Differences in white matter tract K_{trans} between neurological long COVID (NLCV) and cognitively normal (CN) participants (adjusted for age and sex).

**Tract**	**CN** *N*=49	**NLCV** *N*=27	**Group difference** [a]
Corpus callosum	0.00051±0.00021	0.00077±0.00029	**F(1,72)=12.17, p<0.001**
Forceps major	0.00057±0.00027	0.00075±0.00030	F(1,72)=4.34, p=0.04
Forceps minor	0.00033±0.00026	0.00060±0.00028	**F(1,72)=10.74, p=0.002**
Anterior thalamic radiation	0.00025±0.00019	0.00043±0.00025	F(1,72)=7.03, p=0.01
Cortical spinal tract	0.00043±0.00020	0.00063±0.00023	F(1,72)=9.05, p=0.004
Cingulum	0.00013±0.00015	0.00025±0.00017	F(1,72)=5.31, p=0.02
Parahippocampal cingulum	0.00026±0.00020	0.00027±0.00019	F(1,72)=0.04, p=0.84
Fornix	0.00048±0.00030	0.00068±0.00041	F(1,72)=3.52, p=0.06
Inferior frontal occipital	0.00035±0.00015	0.00050±0.00018	**F(1,72)=9.25, p=0.003**
Inferior-frontal superior-frontal	0.00041±0.00019	0.00068±0.00029	**F(1,72)=14.77, p<0.001**
Inferior longitudinal	0.00034±0.00016	0.00051±0.00018	**F(1,72)=10.80, p=0.002**
Superior corticostriatal	0.00043±0.00018	0.00061±0.00024	F(1,72)=7.71, p=0.007
Superior-inferior frontal	0.00035±0.00020	0.00056±0.00024	**F(1,72)=10.28, p=0.002**
Superior longitudinal	0.00041±0.00018	0.00059±0.00022	F(1,72)=8.90, p=0.004
Uncinate	0.00032±0.00019	0.00050±0.00020	F(1,72)=8.47, p=0.005

[a] Bold indicates significant after correction for multiple comparisons (p<0.003).

Supplementary Table 3. Differences in white matter tract free water between neurological long COVID (NLCV) and cognitively normal (CN) participants (adjusted for age and scanner software), are presented for women and men, along with sex by group interactions.

**Tract**	**WOMEN**		**MEN**		**Sex interaction**
	**CN** *N*=25	**NLCV** *N*=21	**CN** *N*=24	**NLCV** *N*=6	
Corpus callosum	-0.19±1.02	0.37±1.09*	-0.09±0.93	-0.16±0.33	*F*(1,71)=2.04, *p*=0.16
Forceps major	0.09±1.22	0.14±0.80	-0.16 ±0.91	-0.25±0.52	*F*(1,71)=0.07, *p*=0.79
Forceps minor	-0.27±1.03	0.47±1.16**	-0.04±0.83	-0.36±0.30	***F*(1,71)=5.44, *p*=0.02**
Anterior thalamic radiation	**-0.41±0.94**	**0.51±0.98****	0.09±1.05	-0.41±0.41	***F*(1,71)=9.95, *p*=0.002**
Cortical spinal tract	-0.04±1.16	0.19±0.99	-0.06±0.83	-0.27±0.67	*F*(1,71)=0.75, *p*=0.39
Cingulum	-0.05±1.00	0.65±1.10*	-0.36±0.88	-0.64±0.45	*F*(1,71)=3.75, *p*=0.06
Parahippocampal cingulum	-0.27 ±1.07	0.29±0.86	0.11 ±1.05	-0.37±0.70	*F*(1,71)=4.03, *p*=0.048
Fornix	-0.40±0.84	0.20±1.10**	0.17±0.85	0.26±0.80	*F*(1,71)=1.86, *p*=0.18
Inferior frontal occipital	-0.33±1.03	0.50±1.05**	0.00±0.93	-0.38±0.52	***F*(1,71)=7.05, *p*=0.01**
Inferior-frontal superior-frontal	-0.18±0.84	0.56±1.25**	-0.19±0.99	-0.43±0.28	*F*(1,71)=3.91, *p*=0.05
Inferior longitudinal	-0.29±1.00	0.41±1.17*	0.03±0.84	-0.34±0.66	***F*(1,71)=4.89, *p*=0.03**
Superior corticostriatal	-0.19±0.77	0.44±1.15*	-0.05±1.11	-0.52±0.44	***F*(1,71)=4.74, *p*=0.03**
Superior-inferior frontal	**-0.32±0.81**	**0.67±1.16*****	-0.13±1.06	-0.52±0.30	***F*(1,71)=8.57, *p*=0.005**
Superior longitudinal	-0.13±0.78	0.46±1.22*	-0.14±1.05	-0.50±0.45	*F*(1,71)=3.47, *p*=0.07
Uncinate	-0.39±0.94	0.65±1.12**	-0.07±0.89	-0.73±0.69	***F*(1,71)=11.15, *p*=0.001**

* *p*<0.05, ** *p*<0.01, *** *p*<0.001 between NLCV and CN

Bold indicates significant after correction for multiple comparisons (*p*<0.003).

Supplementary Table 4. Results of sensitivity analyses of K_{trans} and RSI metrics (adjusted for age and sex) that showed significant group differences in the full sample, using a subset of cognitively normal (CN) participants more closely matched in age and sex to neurological long COVID (NLCV) participants.

**Metric**	**CN** *N*=27	**NLCV** *N*=27	**Group difference** [a]
Gray matter K_{trans}	0.00098±0.00038	0.00134±0.00042	***F*(1,50)=8.67, *p*=0.005**
White matter K_{trans}	0.00041±0.00019	0.00062±0.00024	***F*(1,50)=10.03, *p*=0.003**
Nucleus accumbens K_{trans}	0.00014±0.00014	0.00031±0.00019	***F*(1,50)=9.60, *p*=0.003**
Amygdala neurite density [b]	0.53±0.92	-0.39±0.98	***F*(1,50)=10.19, *p*=0.002**

[a] Bold indicates significant after correction for multiple comparisons

[b] Additionally adjusted for scanner software version

Supplementary Table 5. Linear regression results (standardized *β*, adjusted for age, sex, education, and scanner software) are presented for significant associations between cognitive and RSI measures for either neurological long COVID (NLCV) or cognitively normal (CN), along with group interactions.

**Cognitive measure**	**RSI measure**	**CN** *N*=49	**NLCV** *N*=27	**Group interaction**
Semantic fluency	Gray matter hindered	*β*=0.29, *p*=0.09	***β*=0.71, *p*=0.005**	*β*=0.03, *p*=0.80
Semantic fluency	Gray matter free water	***β*=-0.37, *p*=0.03**	***β*=-0.66, *p*=0.02**	*β*=0.06, *p*=0.59
Semantic fluency	White matter neurite density	*β*=0.13, *p*=0.36	***β*=0.53, *p*=0.03**	*β*=0.11, *p*=0.35
CVLT-II learning	Hippocampal hindered	*β*=0.16, *p*=0.27	***β*=0.63, *p*=0.007**	*β*=0.17, *p*=0.11
CVLT-II learning	Hippocampal free water	*β*=-0.25, *p*=0.11	***β*=-1.03, *p*<0.001**	*β*=-0.21, *p*=0.07
CVLT-II immediate recall	Gray matter hindered	*β*=0.17, *p*=0.33	***β*=0.64, *p*=0.01**	*β*=0.12, *p*=0.30
CVLT-II immediate recall	Hippocampal hindered	*β*=0.31, *p*=0.04	***β*=0.70, *p*=0.002**	*β*=0.12, *p*=0.27
CVLT-II immediate recall	Hippocampal free water	***β*=-0.42, *p*=0.007**	***β*=-1.11, *p*<0.001**	*β*=-0.15, *p*=0.17
CVLT-II delayed recall	Hippocampal free water	***β*=-0.46, *p*=0.003**	***β*=-0.90, *p*=0.002**	*β*=-0.10, *p*=0.38
Trails B	Gray matter restricted isotropic	***β*=-0.28, *p*=0.045**	*β*=0.13, *p*=0.55	***β*=0.23, *p*=0.05**
Trails B	White matter restricted isotropic	***β*=-0.32, *p*=0.03**	*β*=0.02, *p*=0.94	*β*=0.17, *p*=0.13
Trails B	White matter hindered	***β*=0.38, *p*=0.006**	*β*=-0.10, *p*=0.62	***β*=0.26, *p*=0.02**

Bold indicates significant (*p*<0.05, or *p*<0.008 after multiple comparisons correction for subcortical regions).

Supplementary Table 6. Sex-stratified linear regression results (standardized *β*, adjusted for age, education, and scanner software) among neurological long COVID, are presented for significant associations between cognitive and RSI measures for either sex, along with sex interactions.

Cognitive measure	RSI measure	Women *N*=21	Men *N*=6	Sex interaction
Trails B	White matter free water	**β=0.68, p=0.03**	β=-0.09, p=0.85	β=-0.13, p=0.71
Semantic fluency	Gray matter hindered	**β=0.72, p=0.008**	β=0.68, p=0.51	β=0.06, p=0.80
Semantic fluency	Gray matter free water	**β=-0.78, p=0.008**	β=0.74, p=0.65	β=0.27, p=0.24
Semantic fluency	White matter neurite density	**β=0.74, p=0.01**	β=0.44, p=0.58	β=-0.09, p=0.72
Semantic fluency	White matter free water	**β=-0.71, p=0.02**	β=0.10, p=0.90	β=0.14, p=0.70
CVLT-II learning	Hippocampal free water	**β=-1.11, p=0.003**	β=-0.17, p=0.73	β=0.11, p=0.60
CVLT-II immediate recall	Gray matter hindered	**β=0.61, p=0.03**	β=0.70, p=0.21	β=-0.17, p=0.45
CVLT-II immediate recall	Hippocampal free water	**β=-1.22, p<0.001**	β=-0.20, p=0.76	β=0.30, p=0.09
CVLT-II delayed recall	Hippocampal free water	**β=-1.06, p=0.005**	β=0.16, p=0.75	β=0.28, p=0.16

Bold indicates significant (p<0.05, or p<0.008 after multiple comparisons correction for subcortical regions).

Supplementary Table 7. PHS-stratified linear regression results (standardized *ß*, adjusted for age, sex and education) among neurological long COVID, are presented for significant associations between cognitive and K_{trans} or RSI (additionally adjusted for scanner software) measures for low and high polygenic hazard score (PHS), along with PHS interactions.

**Cognitive measure**	**Brain measure**	**Low PHS** *N*=10	**High PHS** *N*=17	**PHS interaction**
Trails B	Gray matter K_{trans}	***ß*=0.63, *p*=0.03**	*ß*=0.16, *p*=0.79	*ß*=-0.35, *p*=0.11
CVLT-II learning	Caudate K_{trans}	*ß*=-0.21, *p*=0.64	***ß*=-1.02, *p*=0.004**	*ß*=-0.38, *p*=0.13
CVLT-II learning	White matter K_{trans}	*ß*=0.24, *p*=0.62	***ß*=-1.27, *p*=0.04**	*ß*=-0.24, *p*=0.39
CVLT-II immediate recall	Caudate K_{trans}	*ß*=-0.12, *p*=0.80	***ß*=-1.07, *p*=0.001**	***ß*=-0.50, *p*=0.03**
CVLT-II delayed recall	Caudate K_{trans}	*ß*=-0.04, *p*=0.91	***ß*=-1.08, *p*=0.002**	*ß*=-0.40, *p*=0.10
CVLT-II delayed recall	White matter K_{trans}	*ß*=0.51, *p*=0.23	***ß*=-1.34, *p*=0.04**	*ß*=-0.25, *p*=0.35
Trails B	White matter free water	***ß*=0.85, *p*=0.007**	*ß*=0.36, *p*=0.62	*ß*=-0.33, *p*=0.10
Semantic fluency	Gray matter restricted isotropic	***ß*=-0.68, *p*=0.03**	*ß*=0.29, *p*=0.64	*ß*=-0.42, *p*=0.31
Semantic fluency	White matter neurite density	***ß*=0.74, *p*=0.03**	*ß*=0.14, *p*=0.89	*ß*=-0.35, *p*=0.16
CVLT-II learning	White matter hindered	*ß*=0.05, *p*=0.89	***ß*=0.82, *p*=0.048**	*ß*=0.57, *p*=0.11

Bold indicates significant (*p*<0.05 or *p*<0.008 after multiple comparisons correction for subcortical regions).

Supplementary Figure 1. **Flow chart of participant inclusion.** Participant sample sizes are presented for each study inclusion and exclusion step, separately for cognitively normal and neurological long COVID participants recruited from the University of California (UCSD) Shiley-Marcos Alzheimer's Disease Research Center (ADRC) and the San Diego community.

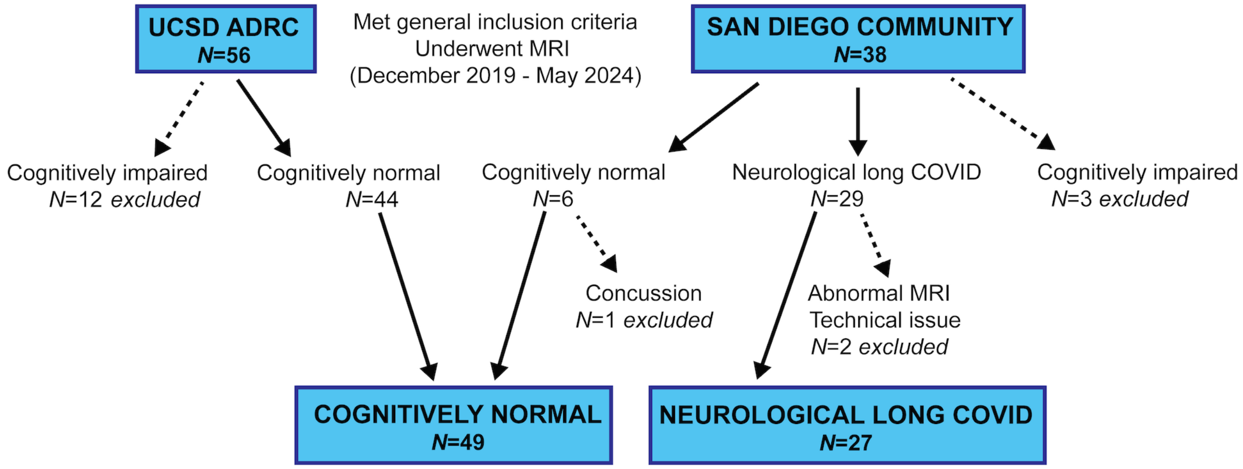

Supplementary Figure 2. **Prevalence and severity of long COVID symptoms.** The prevalence (left) and severity (right) of 23 long COVID symptoms were assessed via self-report. Prevalence refers to the proportion of neurological long COVID participants endorsing each symptom as novel since SARS-CoV-2 infection. Severity ratings are on a scale of 0 (none) to 10 (severe), with error bars representing the standard error.

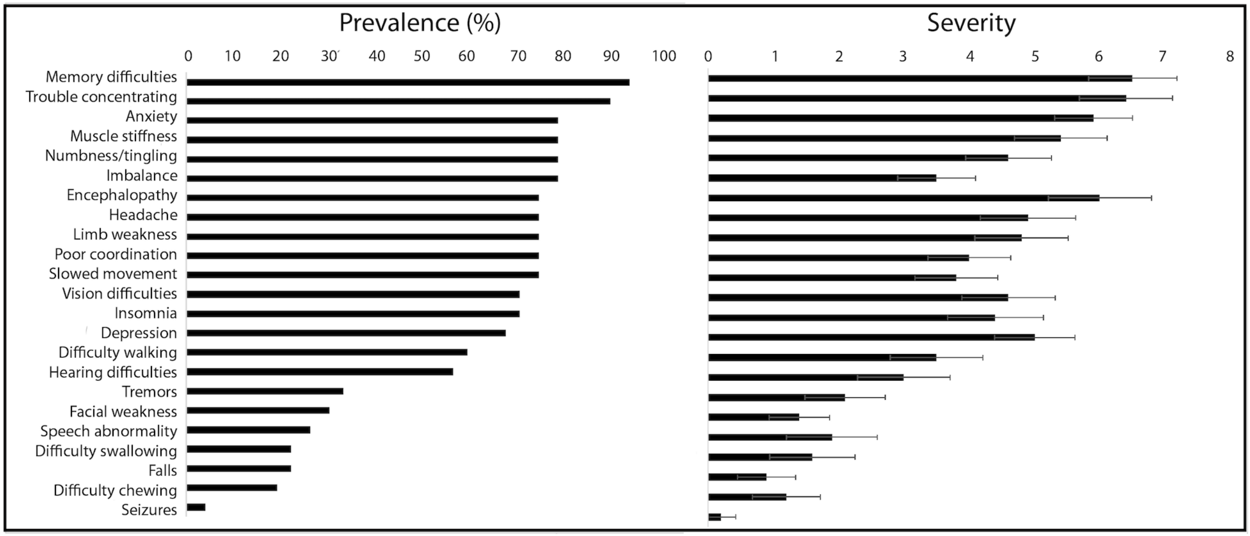

Supplement: Supplementary Material [file imag.a.23_supp.pdf]
